# *PLIN1* Haploinsufficiency Is Not Associated With Lipodystrophy

**DOI:** 10.1210/jc.2017-02662

**Published:** 2018-07-17

**Authors:** Thomas W Laver, Kashyap A Patel, Kevin Colclough, Jacqueline Curran, Jane Dale, Nikki Davis, David B Savage, Sarah E Flanagan, Sian Ellard, Andrew T Hattersley, Michael N Weedon

**Affiliations:** 1 Institute of Biomedical & Clinical Science, University of Exeter, Exeter, United Kingdom; 2 Department of Molecular Genetics, Royal Devon & Exeter NHS Foundation Trust, Exeter, United Kingdom; 3 Department of Endocrinology, Princess Margaret Hospital, Perth, Western Australia, Australia; 4 The Dudley Group NHS Foundation Trust, Dudley, United Kingdom; 5 University Hospital Southampton NHS Foundation Trust, Southampton, United Kingdom; 6 The University of Cambridge Metabolic Research Laboratories, Wellcome Trust-MRC Institute of Metabolic Science, Cambridge, United Kingdom; 7 The National Institute for Health Research Cambridge Biomedical Research Centre, Cambridge, United Kingdom

## Abstract

**Context:**

Monogenic partial lipodystrophy is a genetically heterogeneous disease where only variants with specific genetic mechanisms are causative. Three heterozygous protein extending frameshift variants in *PLIN1* have been reported to cause a phenotype of partial lipodystrophy and insulin resistance.

**Objective:**

We investigated if null variants in *PLIN1* cause lipodystrophy.

**Methods:**

As part of a targeted sequencing panel test, we sequenced *PLIN1* in 2208 individuals. We also investigated the frequency of *PLIN1* variants in the gnomAD database, and the type 2 diabetes knowledge portal.

**Results:**

We identified 6/2208 (1 in 368) individuals with a *PLIN1* null variant. None of these individuals had clinical or biochemical evidence of overt lipodystrophy. Additionally, 14/17,000 (1 in 1214) individuals with *PLIN1* null variants in the type 2 diabetes knowledge portal showed no association with biomarkers of lipodystrophy. *PLIN1* null variants occur too frequently in gnomAD (126/138,632; 1 in 1100) to be a cause of rare overt monogenic partial lipodystrophy.

**Conclusions:**

Our study suggests that heterozygous variants that are predicted to result in *PLIN1* haploinsufficiency are not a cause of familial partial lipodystrophy and should not be reported as disease-causing variants by diagnostic genetic testing laboratories. This finding is in keeping with other known monogenic causes of lipodystrophy, such as *PPARG* and *LMNA*, where only variants with specific genetic mechanisms cause lipodystrophy.

## Teaser

PLIN1 frameshift variants were reported to cause lipodystrophy. We investigated sequencing data from over 140,000 people and established that null variants in PLIN1 do not cause lipodystrophy.

Lipodystrophies are rare disorders characterized by a paucity of subcutaneous fat typically associated with metabolic complications including insulin resistance, diabetes mellitus (DM), hyperandrogenism in females, fatty liver disease, and dyslipidemia (1–4).

Heterozygous protein extending frameshift variants in *PLIN1* have been reported to cause a phenotype of partial lipodystrophy and insulin resistance (1). *PLIN1* encodes perilipin1, a lipid droplet coat protein predominantly expressed in adipocytes. Gandotra *et al.* (1) identified two heterozygous protein-extending frameshift variants in *PLIN1* in three families with partial lipodystrophy and insulin-resistant diabetes. Kozusko *et al*. (5) reported a different heterozygous protein-extending frameshift variant in two families with a similar phenotype.

With the decreasing cost of DNA sequencing, diagnostic genetic testing no longer focuses on screening individual genes but assesses panels of genes or full exomes in a single test. This type of test is extremely useful for genetically heterogeneous conditions such as lipodystrophy. This less selective approach relies on the correct understanding from the literature of which variants within the tested genes are truly pathogenic.

As part of a routine gene panel test for genes reported to be causative of lipodystrophy and monogenic diabetes, we sequenced *PLIN1* in 1323 patients referred for Maturity Onset Diabetes of the Young (MODY) testing. We subsequently studied a further 885 patients with neonatal diabetes or hyperinsulinism who were tested using the same targeted gene panel. We also investigated the frequency of *PLIN1* null variants in 138,632 people in gnomAD (6) and lipodystrophy and diabetes phenotypes in 17,000 individuals in the diabetes knowledge portal. Only three specific *PLIN1* variants have been reported to cause lipodystrophy; our aim was to investigate if null variants in the gene were associated with the same phenotype.

## Materials and Methods

### Recruitment and phenotyping of cohort

At the Royal Devon and Exeter Hospital, 2208 patients were referred for genetic testing to the Molecular Genetics Laboratory. We initially studied 1323 patients with a clinical suspicion of MODY. Subsequently, we studied a further 410 patients with hyperinsulinemic hypoglycemia and 475 patients with neonatal diabetes. Clinical information was provided on a standardized referral form by the clinician at the time of referral for genetic testing, and additional follow-up was carried out for four families. At follow-up, the patients and family members were assessed based on the multisociety practice guidelines on diagnosing lipodystrophy (7). These guidelines provide the list of clinical features that increase suspicion of lipodystrophy. The list contains the following features: generalized or regional absence of body fat, failure to thrive (infants and children), prominent muscles, prominent veins, severe acanthosis nigricans, eruptive xanthomata, cushingoid/acromegaloid/progeroid appearance, DM with high insulin requirements (≥200 U/d or ≥2 U/kg/d or requiring U-500 insulin), severe hypertriglyceridemia (≥500 mg/dL with or without therapy or ≥250 mg/dL despite diet and medical therapy), history of acute pancreatitis secondary to hypertriglyceridemia, nonalcoholic steatohepatitis in a nonobese individual, early-onset cardiomyopathy, and polycystic ovary syndrome.

An insulin measurement was obtained for the father of proband 1 using an Access Ultrasensitive Insulin assay run on a Beckman Coulter Unicel DXI 800 Access Immunoassay System (normal range, 13.0 to 161). Proband 4 had their insulin measured using an Abbott Architect insulin reagent kit on an Abbott Architect i2000SR (reference <83 pmol/L). Reference ranges for the assays were obtained from the laboratories that performed the assays.

A dual-energy x-ray absorptiometry (DEXA) scan was performed on proband 4. This measures the fat distribution within the body and allows the calculation of a fat mass ratio—the ratio between the fat mass of the trunk and the lower limbs (8).

### Genetic analysis

We sequenced *PLIN1* (NM_002666.4) by targeted next generation sequencing, as part of a gene panel test (methodology described previously) (9). The panel contains baits for genes in which pathogenic variants are reported to cause a range of pancreatic-linked phenotypes (including lipodystrophy, monogenic diabetes, and hyperinsulinism); only those relevant to the patient’s disease are reported. Each sample was sequenced with a minimum of 3.3 million reads and greater than 99% of targeted bases covered at 30× or higher. All *PLIN1* variants were also confirmed by Sanger sequencing.

## Results

### The phenotype of patients with heterozygous null variants in *PLIN1* is not consistent with overt lipodystrophy

**Figure 1.**
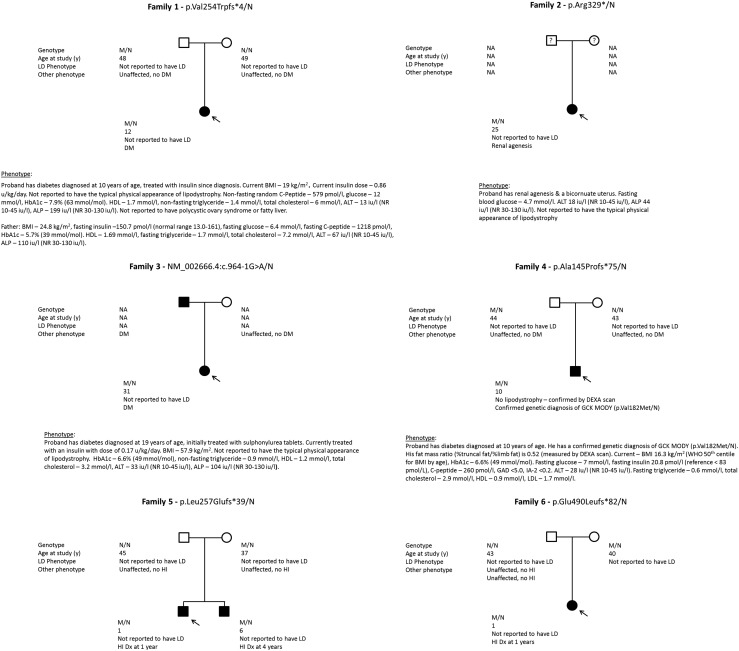
Pedigrees of the families with *PLIN1* null variants. ALP, alkaline phosphatase; ALT, alanine transaminase; BMI, body mass index; Dx, diagnosed; GAD, glutamic acid decarboxylase autoantibodies; GCK, glucokinase; HbA1c, glycated hemoglobin; HI, hyperinsulinemic hypoglycemia; HLD, high-density lipoprotein; LD, lipodystrophy; LDL, low-density lipoprotein; NR, normal range; WHO, World Health Organization.

We initially tested 1323 patients with a clinical suspicion of MODY of which four had a heterozygous null variant in *PLIN1* (probands 1 to 4; Table 1 and Fig. 1). We investigated data from a further 885 non-MODY patients sequenced with the same genetic panel test, two of whom had a heterozygous null variant in *PLIN1* (probands 5 and 6; Table 1 and Fig. 1). All of these variants are nonsense or frameshift variants. None of these patients had symptoms of lipodystrophy. The published patients with pathogenic *PLIN1* variants had dyslipidemia with hypertriglyceridemia (range from 2 to 147 mmol/L) (1, 5); all of the patients in our study with variants in *PLIN1* had triglyceride levels below 1.7 mmol/L. Proband 4, who has a genetic diagnosis of *GCK* MODY due to a heterozygous pathogenic missense variant (p.Val182Met), had a fasting glucose of 7 mmol/L, fasting insulin of 20.8 pmol/L, and had a DEXA scan showing his fat mass ratio is 0.52. Valerio *et al.* (8) used DEXA to measure the fat mass ratio of a cohort of patients with partial lipodystrophy and showed that their mean ratio was 1.86 compared with controls with a mean ratio of 0.93. However, although these results demonstrate that the phenotype of proband 4 is inconsistent with lipodystrophy, he is only 10 years of age thus features could present later and DEXA scans at this age cannot conclusively exclude lipodystrophy.

**Table 1.** Details of *PLIN1* Variants and Phenotypic Data for the Probands and Family Members in Our Cohort

Family ID	1		2	3	4		5			6	
Relation	Proband	Father	Proband	Proband	Proband	Father	Proband	Brother	Mother	Proband	Mother
Sex	Female	Male	Female	Female	Male	Male	Male	Male	Female	Female	Female
Age at study, y	12	48	25	31	10	44	1	6	37	1	40
Ethnicity	Asian (Filipino), UK	Asian (Filipino), UK	Mixed (White and Black African), UK	White, UK	White, Australia	White, Australia	Arabic, Saudi Arabia	Arabic, Saudi Arabia	Arabic, Saudi Arabia	White, Austria	White, Austria
c.Nomen	NM_002666.4:c.760del	NM_002666.4:c.760del	NM_002666.4:c.985C>T	NM_002666.4:c.964-1G>A	NM_002666.4:c.433del	NM_002666.4:c.433del	NM_002666.4:c.768_770delinsTG	NM_002666.4:c.768_770delinsTG	NM_002666.4:c.768_770delinsTG	NM_002666.4:c.1448_1466dup	NM_002666.4:c.1448_1466dup
p.Nomen	p.Val254Trpfs*4	p.Val254Trpfs*4	p.Arg329*	p.?	p.Ala145Profs*75	p.Ala145Profs*75	p.Leu257Glufs*39	p.Leu257Glufs*39	p.Leu257Glufs*39	p.Glu490Leufs*82	p.Glu490Leufs*82
Exon	6	6	8	8	5	5	6	6	6	9	9
Diagnosis	DM	Unaffected	Renal agenesis	DM	DM	Unaffected	HI	HI	Unaffected	HI	Unaffected

Abbreviation: HI, hyperinsulinemic hypoglycemia.

Symptoms of familial partial lipodystrophy typically present at puberty in women. Two of our probands were over 18 years old and did not display the characteristic fat displacement or have biochemical evidence of overt lipodystrophy. Additionally, family member testing for four probands identified the *PLIN1* variant in an unaffected parent and one sibling (Table 1). Three of the parents had no overt symptoms of lipodystrophy at the ages of 40, 44, and 46, and although the father of proband 1, 48 years old, showed impaired fasting glycemia with high cholesterol level but normal fasting triglyceride and high high-density lipoprotein levels, these results are not consistent with severe overt lipodystrophy.

### Null variants in *PLIN1* were not associated with biomarkers of lipodystrophy in an independent cohort

The lack of an overt lipodystrophy phenotype in patients with null variants in *PLIN1* raised questions over the pathogenicity of protein truncating variants in this gene. We used the type 2 diabetes knowledge portal to assess the association of *PLIN1* to the biomarkers of lipodystrophy (10). The type 2 diabetes knowledge portal is a repository of 17,000 people who have been exome sequenced and had certain phenotypes tested. There are seven different *PLIN1* null variants in 14 people, giving a frequency of 1 in 1214. *PLIN1* null variants were not associated with higher fasting insulin, higher triglyceride, higher low-density lipoprotein, or lower high-density lipoprotein (Supplemental Table 1), indicating that these individuals do not have overt lipodystrophy.

### Null variants in *PLIN1* are present at a frequency in gnomAD which is higher than expected

In the publically available gnomAD database, 126/138,632 (1 in 1100) people have heterozygous null variants (6). *PLIN1* is not well covered in all individuals in gnomAD, so the true frequency could be even higher (see Supplemental Fig. 1). The pLI constraint score for loss of function intolerance is 0 for *PLIN1*, predicting that these variants are tolerated based on their frequency (6). These findings are not consistent with heterozygous null variants causing overt lipodystrophy. However, there are no null variants in a homozygous state in gnomAD.

## Discussion

### *PLIN1* variants are unlikely to be pathogenic via a mechanism of haploinsufficiency

We present six probands and five family members who have null variants in *PLIN1* but no phenotype of lipodystrophy. Importantly, six of these individuals are over 18 years old, when familial partial lipodystrophy is expected to have presented, and four are over the age of 40 years old. We were able to obtain follow-up information for four families. Although it was not possible to obtain full information on all of these individuals, we have clear biochemical measures, such as triglycerides, for several individuals demonstrating they are unlikely to have severe lipodystrophy at the time of study (however, proband 4 was only 10 years of age; thus, there is the potential to develop symptoms at a later date). Another limitation of our study is that all biochemistry parameters were measured locally with different commercial assays. We have limited information in our original cohorts on all the features of lipodystrophy that have been proposed by the multisociety practice guidelines (7). However, this is not a concern in our study as all of the patients in the cohort had undergone genetic testing for all the genes on our panel, irrespective of their clinical features. This robust analysis has made sure that we did not miss any patients with variants in *PLIN1*. It is possible that although *PLIN1* heterozygous null variants do not appear to cause the overt lipodystrophic phenotype described in Gandotra *et al*. (1), they may cause a more subtle form of the disease.

Familial partial lipodystrophy is estimated to have a prevalence of approximately 3 in 1 million in the general population (11). The frequency of null variants in the gnomAD database is incompatible with the frequency of lipodystrophy. The prevalence of familial partial lipodystrophy would need to be greater than 1 in 10,000 to be consistent with null variants in *PLIN1* causing the disorder (12). If these variants caused familial partial lipodystrophy, then the disease would be expected to affect 60,000 people in the United Kingdom. Additionally, the type two knowledge portal provides a set of individuals with a high frequency of *PLIN1* variants where there is no association with raised insulin or lipids. Thus, variants in *PLIN1* are unlikely to be acting via a mechanism of true haploinsufficiency, *i.e.*, complete lack of one allele.

### *PLIN1* is an example of a gene where only variants with specific genetic mechanisms are likely to be pathogenic

There is a range of genetic mechanisms of pathogenicity for the known monogenic causes of lipodystrophy. For example, some variants in *PPARG* demonstrate dominant negative behavior (2, 3), whereas others appear to be pathogenic due to haploinsufficiency. A different pattern is observed in *LMNA* where haploinsufficiency causes phenotypes including cardiomyopathy, whereas specific missense variants result in lipodystrophy, and other specific mutations in *LMNA* cause distinct phenotypes, including progeria (4).

The *PLIN1* frameshift variants published as pathogenic do not appear in gnomAD. However, it is also possible that the reason we do not see these in gnomAD is that the last exon has very low sequencing coverage: the majority of samples have less than 10× coverage (see Supplemental Fig. 1). The absence of the published variants in gnomAD and our cohort prevents us from commenting on their pathogenicity.

A possible explanation for the high frequency of *PLIN1* null variants in public databases would be that variants are pathogenic but have low penetrance or reduced clinical expressivity. However, given their frequency, they would either need to have very low penetrance or the disease would need to be more common than assumed (more common than 1 in 10,000).

### *PLIN1* variants resulting in haploinsufficiency should not be reported as pathogenic without further evidence

Doubt over haploinsufficiency in *PLIN1* causing lipodystrophy has important consequences for genetic testing. *PLIN1* is currently screened on diagnostic targeted gene panel tests for lipodystrophy, insulin resistance, and diabetes. We recommend that null variants in *PLIN1* should not be reported as causative of lipodystrophy.

Our study suggests that heterozygous null variants in *PLIN1* do not cause overt lipodystrophy and provides another example of a gene where only variants with specific genetic mechanisms cause lipodystrophy.

## Supplementary Material

Supplemental Data

## Abbreviations:

DEXA: dual-energy x-ray absorptiometry
DM: diabetes mellitus
MODY: Maturity Onset Diabetes of the Young
