# Supplementary material for: PLIN1 Haploinsufficiency Is Not Associated With Lipodystrophy
Source: J Clin Endocrinol Metab. 2018 Jul 17;103(9):3225–30. doi: 10.1210/jc.2017-02662 (PMC6126890; doi:10.1210/jc.2017-02662)

**Supplementary Table 1**

Association statistics from the Type 2 diabetes knowledge portal (<http://www.type2diabetesgenetics.org/gene/geneInfo/PLIN1>) for a range of diabetes and lipid phenotypes. In total there were 7 variants reported as protein truncating in 14 people in this dataset.

| **Phenotype** | **N** | **Odds Ratio** | **beta** | ***P*** | **95% CI** |
| --- | --- | --- | --- | --- | --- |
| Type 2 diabetes | 8466 cases /  8373 controls | 0.469 | NA | 0.209 | 0.144 to 1.53 |
| BMI | 15926 | NA | -0.902 | 0.541 | -3.79 to 1.99 |
| Fasting glucose | 10991 | NA | 0.325 | 0.0889 | -0.0494 to 0.700 |
| Fasting insulin | 8102 | NA | -0.31 | 0.474 | -1.16 to 0.538 |
| Cholesterol | 14233 | NA | 0.0896 | 0.782 | -0.543 to 0.723 |
| LDL Cholesterol | 12355 | NA | -0.0458 | 0.884 | -0.663 to 0.571 |
| HDL Cholesterol | 13264 | NA | 0.216 | 0.071 | -0.0184 to 0.450 |
| Triglycerides | 14225 | NA | -0.0819 | 0.609 | -0.396 to 0.232 |

**Supplementary Figure 1**

Proportion of samples which have a coverage depth of 10x across *PLIN1* in gnomAD (samples sequenced as exomes shown by the blue areas on the graph representing exons 1-8, green lines representing the samples sequenced as genomes). With the positions shown beneath of the variants from gnomAD, this study and those previously published to cause lipodystrophy (variants published by Gandotra *et al.* 2011 marked in blue and the variant publisbed by Kozusko *et al.* 2015 marked in pink).


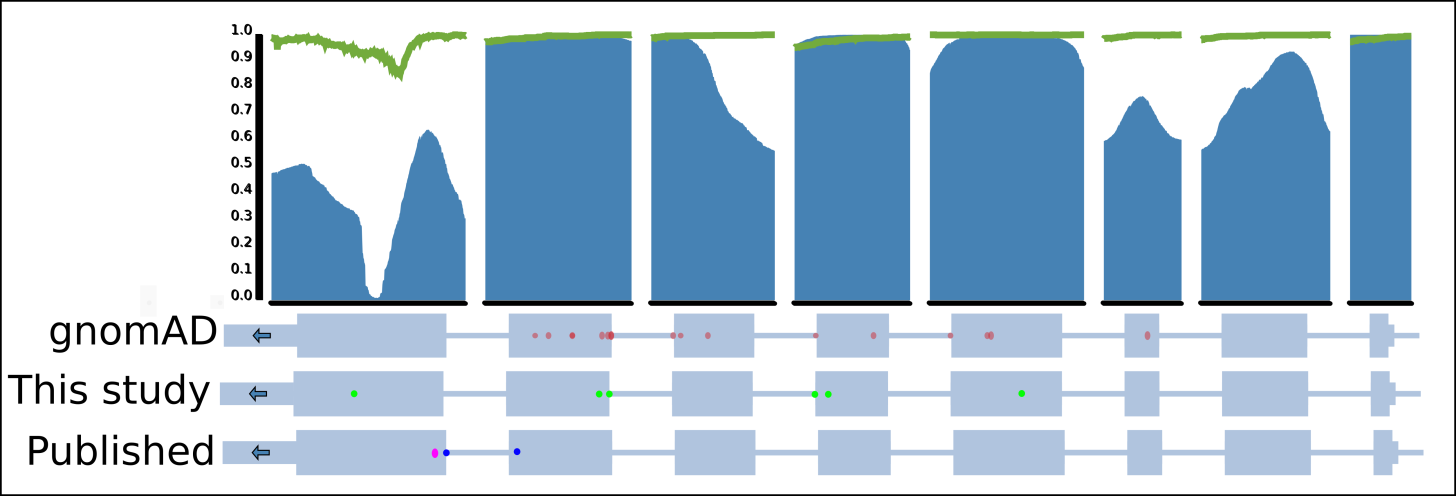

Supplement: Supplemental Data [file jc.2017-02662.sd1.docx]
